# The relationship between pituitary size and the response to recombinant human growth hormone therapy in children with isolated growth hormone deficiency

**DOI:** 10.3389/fendo.2026.1762583

**Published:** 2026-02-25

**Authors:** Özge Köprülü, Mehmet Coşkun, Ezgi Çelik, İbrahim Mert Erbaş, Özlem Nalbantoğlu, Hüseyin Anıl Korkmaz, Behzat Özkan

**Affiliations:** 1 Department of Pediatric Endocrinology, University of Health Sciences Izmir Dr. Behçet Uz Children’s Education and Research Hospital, Izmir, Türkiye; 2 Department of Radiology, University of Health Sciences Izmir Dr. Behçet Uz Children’s Education and Research Hospital, Izmir, Türkiye; 3 Department of Pediatrics, University of Health Sciences Izmir Dr. Behçet Uz Children’s Education and Research Hospital, Izmir, Türkiye

**Keywords:** growth hormone deficiency, growth velocity, pituitary height, pituitary size, pituitary volume, recombinant human growth hormone

## Abstract

**Background:**

Growth hormone deficiency (GHD) is one of the major endocrine causes of short stature in childhood. Pituitary size may reflect growth hormone secretory capacity; and children with hypoplastic pituitary exhibit more severe GHD. Given this relationship, pituitary size may also serve as a valuable predictor of the growth response to recombinant human growth hormone (rhGH) therapy. This study aimed to investigate the relationship between pituitary height and volume measured on MRI and the growth response to rhGH therapy in children with GHD.

**Methods:**

This retrospective, single-center study included 52 children with isolated GHD. Pretreatment pituitary MRI was evaluated for pituitary height and volumetric assessment. Two different methods were used to estimate pituitary volume: the classical ellipsoid formula and and the cross-sectional area. Pituitary volume SDS values were calculated according to age- and sex. Growth response to therapy was measured using height velocity (HV), HV SDS, ΔHeight SDS (change in height SDS between baseline and the end of the first year of the treatment), and ΔIGF1 SDS (change in IGF1 SDS between baseline and the end of the first year of the treatment).

**Results:**

The median age of the patients at diagnosis was 8.1 years (IQR: 4.4-11.4). At diagnosis, patients showed marked growth failure with a median height SDS of -2.84 (IQR: −3.65 to −2.44). Statistically significant negative correlation was observed between pituitary height and both ΔHeight SDS and ΔIGF1 SDS. Pituitary volume calculated by the ellipsoid method showed statistically significant negative correlations with HV, HV SDS, and ΔHeight SDS at the end of the first year of rhGH therapy (p < 0.05). Pituitary volume calculated by the cross-sectional area method only showed statistically significant negative correlation with HV SDS. When patients were categorized based on their response to rhGH therapy, those in the good-response group (HV SDS > 2) had significantly lower pituitary volume SDS calculated by the ellipsoid method compared to poor responders (p = 0.021).

**Conclusions:**

Our findings indicate that smaller pituitary size is associated with a more favorable growth response to rhGH therapy in children with isolated GHD.

## 1 Introduction

Growth hormone deficiency (GHD) is a relatively uncommon cause of short stature in the general pediatric population, with an estimated prevalence ranging from 1 in 4,000 to 1 in 10,000 children. However, it constitutes a significant proportion of referrals to pediatric endocrinology clinics, especially in children with growth failure not attributable to familial or constitutional causes (1). Confirming GHD requires combination of clinical features with auxological data, laboratory analysis, endocrine dynamic tests, bone age, and imaging of the hypothalamic–pituitary region (2).

Early and accurate diagnosis of GHD is crucial, as recombinant human growth hormone (rhGH) replacement therapy is highly effective (2, 3). rhGH therapy significantly improves growth velocity and final adult height in children with GHD. However, the growth response varies with age, sex, pubertal status, peak growth hormone (GH) levels in stimulation tests, and mid-parental height (2, 4). The effectiveness of rhGH therapy with respect to growth velocity is greatest in children with organic abnormalities in the hypothalamic–pituitary region, who usually require lower doses of the hormone to achieve a sufficient response (5–7).

Several studies have shown that morphological abnormalities detectable on pituitary magnetic resonance imaging (MRI) such as hypoplastic anterior pituitary, interrupted pituitary stalk, empty sella, and ectopic posterior pituitary are strongly associated with severe GHD (5–12). Previous studies have also demonstrated that pituitary size reflects GH secretory capacity; and children with hypoplastic pituitary exhibit more severe height deficit (13–17). Reduced pituitary height or volume may reflect impaired somatotroph function or a lower number of somatotroph cells (14, 18, 19). Given this relationship, pituitary size may also serve as a valuable predictor of the growth response to rhGH therapy.

This study aimed to investigate the relationship between pituitary height and volume measured on MRI and the growth response to rhGH therapy in children with GHD. Understanding this relationship may help clinicians better predict therapeutic outcomes and develop personalized treatment strategies for children.

## 2 Material methods

### 2.1 Study design and patients

This retrospective, single-center study included children diagnosed with isolated GHD who were followed in the Department of Pediatric Endocrinology, Izmir Dr. Behçet Uz Children’s Hospital between December 2020 and December 2025. A total of 52 patients were enrolled. A structured questionnaire was used to systematically evaluate both clinical, hormonal and radiological data. The Standard Deviation Scores (SDS) of weight, height, and body mass index (BMI) were calculated based on Turkish children’s reference values (20).

Inclusion criteria were:

1. Diagnosis of GHD based on auxological findings, decreased growth velocity, low IGF1 levels, and peak GH <10 μg/L during two separate growth hormone stimulation tests,
2. Available of pre-treatment pituitary MRI obtained before initiation of rhGH therapy,
3. At least 12 months of rhGH therapy with documented follow-up height measurements.

Exclusion criteria were:

1. Chronic systemic diseases or genetic syndromes affecting growth,
2. History of cranial radiotherapy, neurosurgery, or central nervous system tumors,
3. Incomplete clinical or imaging data,
4. Multiple pituitary hormone deficiencies,
5. Organic hypothalamic–pituitary abnormalities such as interrupted pituitary stalk, empty sella, and ectopic posterior pituitary.

All patients received rhGH (somatropin) at an initial dose of 0.035 mg/kg/day, in accordance with standard pediatric GHD treatment guidelines. During the treatment period, no patient showed an IGF1 SDS above +2, and no dose adjustments were necessary. Dosage adjustments during follow-up were based on IGF levels, growth velocity, and clinical response. Growth response to therapy was measured using height velocity (HV), HV SDS, ΔHeight SDS (change in height SDS between baseline and the end of the first year of the treatment), and ΔIGF1 SDS (change in IGF1 SDS between baseline and the end of the first year of the treatment). HV SDS and IGF1 SDS values were calculated according to age- and sex-specific normative data (21).

### 2.2 MRI acquisition and volume measurements

All pituitary MRIs were performed using a 1.5-T system (Philips Ingenia; Philips Medical Systems, Eindhoven, The Netherlands) with a 32-channel head coil. Pituitary MRI protocol included pre-contrast high resolution sagittal T1-weighted imaging (T1-WI), coronal T2WI, dynamic MRI in coronal plane with T1WI sequence, post-contrast sagittal and coronal T1WI. Pre-contrast high resolution sagittal T1WI and coronal T2WI were used in volume calculation. The acquisition parameters of those sequences were as follows: 1. Sagittal T1WI: field-of-view (FOV), 120×120 mm; acquisition matrix, 200×200; slice thickness, 2.5 mm; gap, 0 mm; repetition time (TR), 550 msn; echo time (TE), 14 msn. 2. Coronal T2WI: FOV, 120×120 mm; acquisition matrix, 200×155; slice thickness, 2 mm; gap, 0.2 mm; TR, 2000 msn; TE, 125 msn.

**Figure 1:**
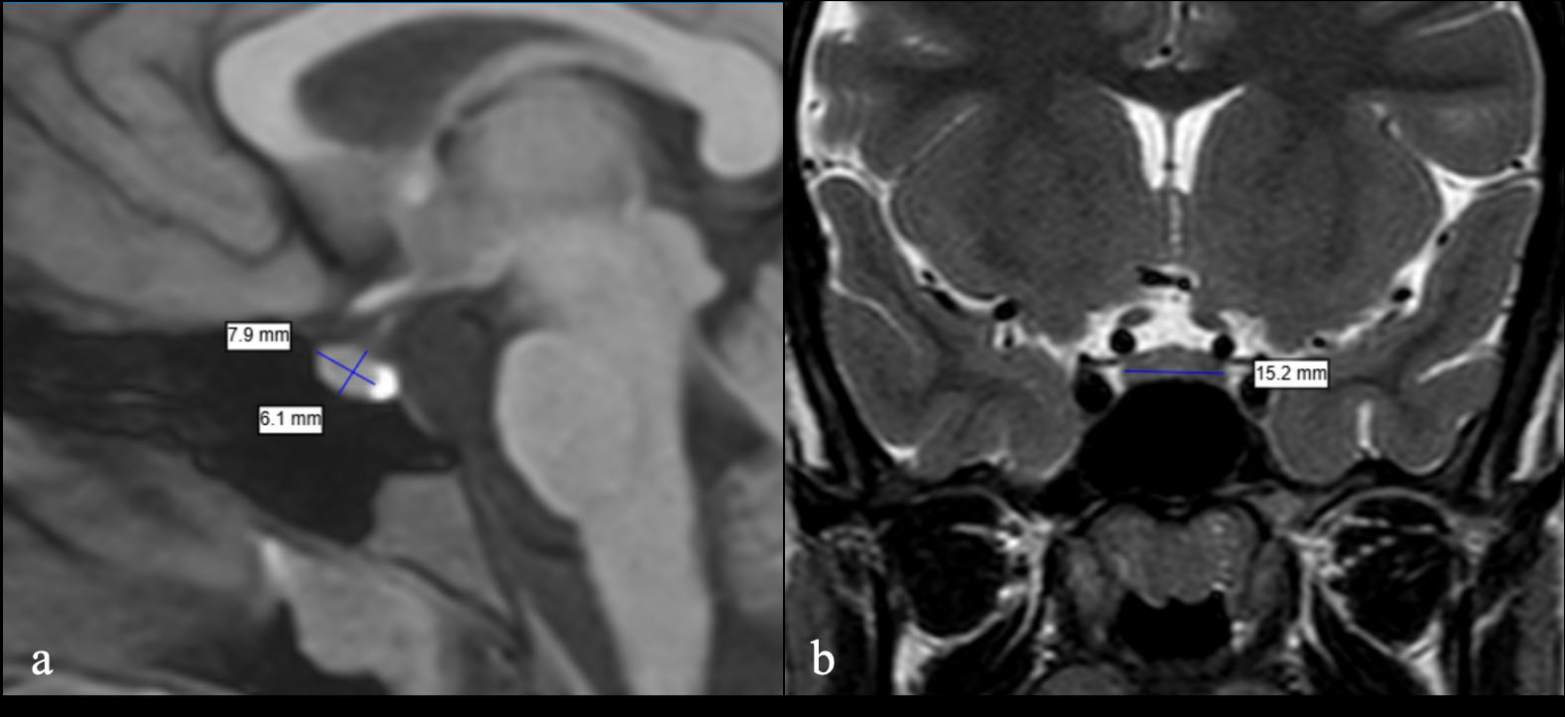
Volume measurement of the adenohypophysis obtained using the ellipsoid formula on high-resolution sagittal T1WI **(a)** and coronal T2WI **(b)**.

The maximum height of the adenohypophysis on the mid-sagittal slice of the high-resolution T1WI was defined as the gland height. For volumetric assessment, only the adenohypophysis was measured; the neurohypophysis was excluded from all calculations. Two different methods were used to estimate pituitary volume:

In the first method (volume 1), the classical ellipsoid formula (product of three orthogonal diameters × 0.52) was used. In this approach, the craniocaudal dimension between the base of the adenohypophysis and the insertion point of the pituitary stalk on the mid-sagittal slice, the anteroposterior length perpendicular to craniocaudal measurement of the adenohypophysis on the same slice, and the intermedial distance between the medial borders of the cavernous sinuses on the mid-coronal slice were measured. These three measurements were multiplied together and then by the coefficient 0.52 to obtain the estimated volume (**Figure 1**).

**Figure 2:**
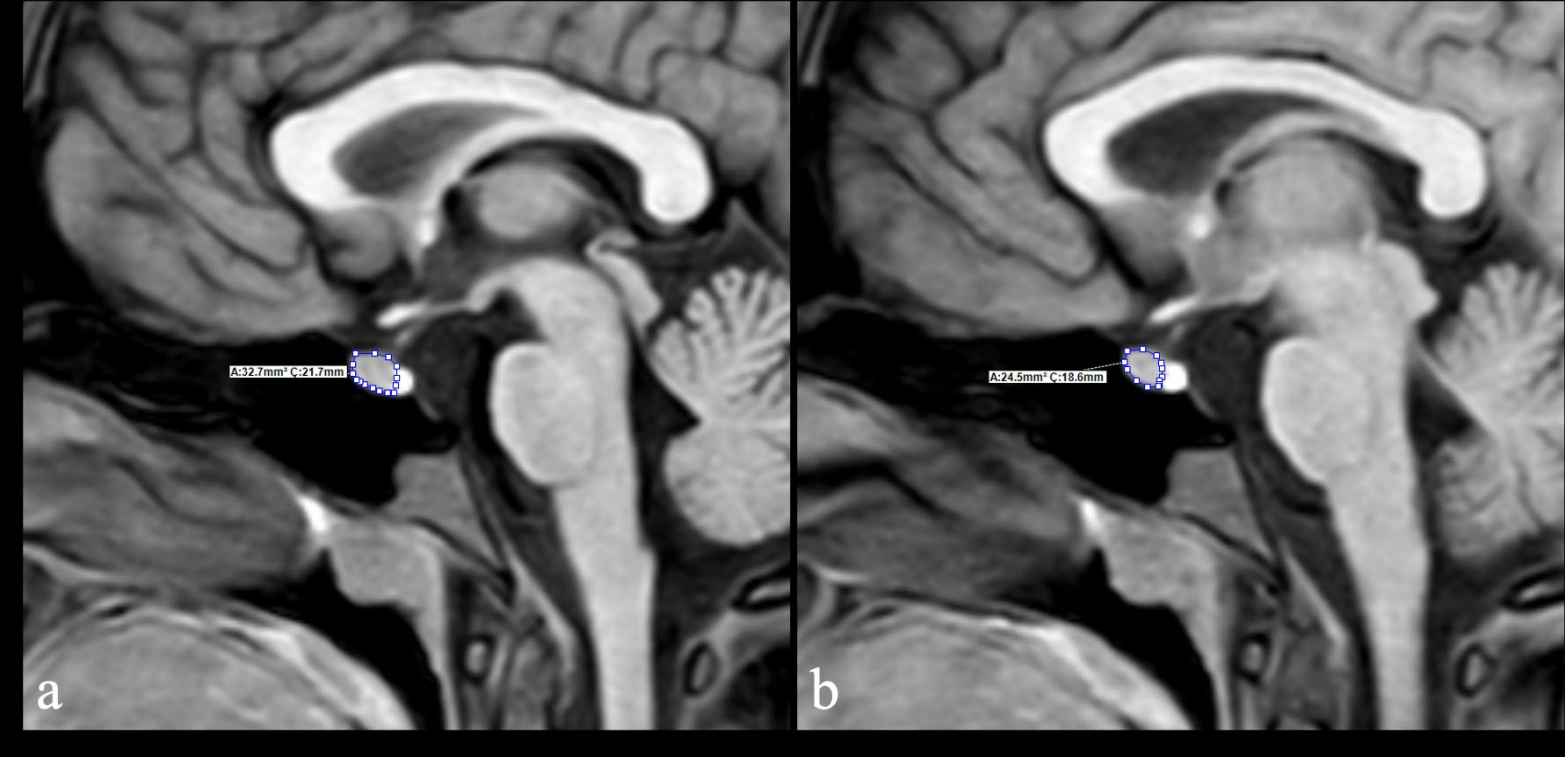
Volume measurement of the adenohypophysis obtained by area tracing across serial high-resolution sagittal T1W slices **(a, b)**.

In the second method (volume 2), on each visible slice of the adenohypophysis on high-resolution sagittal T1WI, the contours of the adenohypophysis were manually traced by a radiologist with 10 years of experience, and the cross-sectional area was calculated. The areas from all slices were summed and multiplied by the slice thickness to obtain the total volume of adenohypophysis (**Figure 2**).

Pituitary volume SDS values were calculated according to age- and sex-specific normative data from a large-scale MRI study of 517 Turkish children (22).

### 2.3 Statistical analysis

SPSS 25.0 (IBM Corporation, Armonk, New York, United States) was used to analyze the variables. Due to the relatively small sample size, the assumption of normality was assessed with both Shapiro–Wilk test and histograms. The Student**’**s T-test or the Mann–Whitney U- tests were used to evaluate the differences between two groups based on the distribution of the parameters. The Chi-square test was used to compare the categorical variables. Quantitative variables were given as the mean (standard deviation) or the median (25th and 75th percentiles). The relationship between pituitary size (height and volume) and growth response variables was analyzed using Pearson and Spearman correlation tests. The p value less than 0.05 was considered significant.

### 2.4 Ethical

The study was conducted in accordance with the Declaration of Helsinki and approved by the Ethics Committee of Dr. Behcet Uz Children’s Hospital (protocol code: 2025/19-14). Informed consent was obtained from all subjects and parents involved in the study. Written informed consent has been obtained from the patients and parents to publish this paper.

## 3 Results

Fifty-two children with isolated GHD were included in the study. Five patients were excluded from the study due to the presence of organic hypothalamic–pituitary abnormalities. The clinical, hormonal, radiological characteristics and treatment outcomes were analyzed to determine the association between pituitary size and growth response to rhGH therapy. The 57.4% (n=27) of the patients were male, and 42.6% (n=20) were female.

The median age at diagnosis was 8.1 years (IQR: 4.4-11.4). At diagnosis, patients showed marked growth failure with a median height SDS of -2.84 (IQR: −3.65 to −2.44), a mean weight SDS of -2.08 ± 1.07, and a mean BMI SDS of -0.34 ± 1.12. The mean mid-parental height SDS was -1.11 ± 0.96. Baseline IGF1 SDS was low, with a median of -2.23 (IQR: −2.89 to −1.48). The mean peak GH values in stimulation with L-Dopa and Clonidine were 2.90 ± 1.77 ng/mL and 4.41 ± 2.67 ng/mL, respectively. The mean duration of rhGH therapy was 19.53 ± 11.33 months (median 16.0, IQR 10.0–25.5). **Table 1** summarizes the clinical and laboratory findings of the patients at the time of diagnosis.

**Table 1:** Baseline characteristics of the patients.

Variables	Mean ± SD	Median (IQR)
Age at baseline (years)	8.07 ± 3.96	8.1 (4.4–11.4)
Height SDS	-2.96 ± 0.93	-2.84 (−3.65 to −2.44)
Weight SDS	-2.08 ± 1.07	-2.28 (−3.02 to −1.28)
BMI SDS	-0.34 ± 1.12	-0.41 (−1.14 to 0.56)
Target height SDS	-1.11 ± 0.96	-1.31 (−1.74 to −0.41)
Baseline IGF1 SDS	-2.14 ± 1.16	-2.23 (−2.89 to −1.48)
Peak GH (L-dopa, µg/L)	2.90 ± 1.77	2.2 (1.4–3.8)
Peak GH (Clonidine, µg/L)	4.41 ± 2.67	3.7 (2.2–5.7)
Treatment duration (months)	19.53 ± 11.33	16.0 (10.0–25.5)
Pituitary height (mm)	4.41 ± 0.97	4.34 (3.75–5.03)
Pituitary volume 1 (ellipsoid, mm³)	148.18 ± 61.22	131.93 (100.73–200.61)
Pituitary volume 1 (SDS)	-0.61 ± 0.91	-0.54 (−0.97 to −0.10)
Pituitary volume 2 (cross-sectional area, mm³)	191.07 ± 89.79	167.2 (122.38–249.48)
Pituitary volume 2 (SDS)	0.07 ± 1.20	-0.02 (−0.39 to 0.73)

BMI, body mass index; SDS, standard deviation score; IQR, interquartile range.

Pituitary MRI performed at diagnosis and revealed a mean mid-sagittal pituitary height of 4.41 ± 0.97 mm (median 4.34, IQR 3.75–5.03). The mean pituitary volume calculated using the ellipsoid formula (volume 1) was 148.18 ± 61.22 mm³ with a median Z score -0.54 SDS (IQR −0.97 to −0.10), whereas volume calculated by the cross-sectional area method (volume 2) was 191.07 ± 89.79 mm³ with a mean Z score 0.07 ± 1.20 SDS. As expected, pituitary height showed a strong positive correlation with both volume measurements (volume 1: r = 0.79; volume 2: r = 0.72).

In the correlation analysis, pituitary height and volume—measured using both the ellipsoid formula and the cross-sectional area method—showed no significant relationship with baseline height SDS or peak GH responses. In contrast, both pituitary height and volume demonstrated a moderate and statistically significant positive correlation with baseline IGF1 SDS (r = 0.607, p < 0.001).

After one-year of rhGH treatment, the mean height velocity (HV) was 9.04 ± 2.95 cm/year (median 8.6, IQR 7.22–10.3). The mean HV SDS was 2.38 ± 2.60 (median 2.00, IQR 0.4–3.57). The mean ΔHeight SDS was 0.80 ± 0.77 (median 0.81, IQR 0.22–1.29), and the mean ΔIGF1 SDS was 1.50 ± 1.66 (median 1.23, IQR 0.30–2.66). Based on height velocity SDS during the first year of therapy, 24 patients were classified as good responders (HV SDS > +2), and 23 patients were classified as poor responders (HV SDS ≤ +2). (**Table 2**)

**Table 2:** Growth response to rhGH therapy for one-year.

Variables	Mean ± SD	Median (IQR)
HV (cm/years)	9.03 ± 2.95	8.6 (7.22–10.3)
HV SDS	2.38 ± 2.60	2.00 (0.4–3.57)
ΔHeight SDS	0.80 ± 0.77	0.81 (0.22–1.29)
ΔIGF1 SDS	1.50 ± 1.66	1.23 (0.30–2.66)

HV, height velocity; SDS, standard deviation score; IQR, interquartile range.

**Figure 3:**
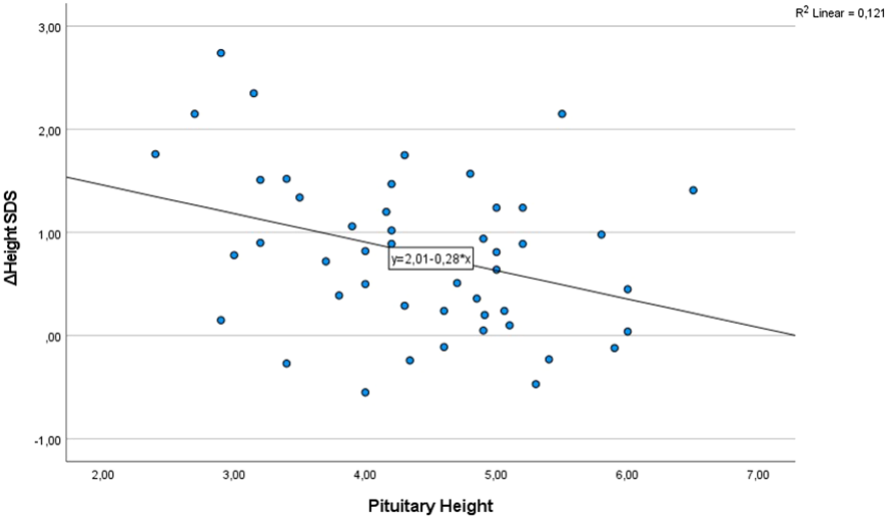
Negative correlation between ΔHeight SDS at the end of the first year and pituitary height (mm).

As shown in **Table 3**, correlation analysis did not demonstrate any significant correlations between pituitary height and height velocity or height velocity SDS at the end of the first year of rhGH therapy. However, a weak but statistically significant negative correlation was observed between pituitary height and both ΔHeight SDS (**Figure 3**) and ΔIGF1 SDS. Pituitary volume calculated by the ellipsoid method showed statistically significant negative correlations with HV, HV SDS, and ΔHeight SDS at the end of the first year of rhGH therapy (p < 0.05 for all). Pituitary volume calculated by the cross-sectional area method only showed statistically significant negative correlation with HV SDS. No significant associations were observed for pituitary volume derived from the cross-sectional area method with HV or ΔHeight SDS. Pituitary volume did not correlate with ΔIGF1 SDS in either method.

**Table 3:** Correlation coefficients (r) between pituitary measurements and growth response after one year of rhGH therapy.

Variables	Correlation metrics	Pituitary height	Pituitary volume 1 SDS (ellipsoid formula)	Pituitary volume 2 SDS (cross-sectional area)
HV	Pearson/Spearman r	-0.266	-0.314*	-0.194
	p	0.070	**0.030**	0.190
HV SDS	Pearson/Spearman r	-0.267	-0.375**	-0.293*
	p	0.060	**0.009**	**0.045**
ΔHeight SDS	Pearson/Spearman r	-0.348*	-0.376**	-0.178
	p	**0.010**	**0.009**	0.232
ΔIGF1 SDS	Pearson/Spearman r	-0.344*	-0.109	-0.128
	p	**0.010**	0.465	0.391

Statistically significant correlations (p<0.05) are highlighted in bold.

*p < 0.05 indicates statistically significant correlation.

**Correlation is significant at the 0.01 level.

HV, height velocity; SDS, standart deviation score.

When patients were categorized based on their response to rhGH therapy, those in the good-response group (HV SDS > 2) had significantly lower pituitary volume SDS calculated by the ellipsoid method compared to poor responders (p = 0.021). No significant differences were found between the groups in terms of pituitary height, pituitary volume (both ellipsoid and cross-sectional area method) or pituitary volume SDS calculated by the cross-sectional area method (**Table 4**).

**Table 4:** Comparison of pituitary height and volume between good and poor responders to rhGH therapy.

Pituitary measurements	Good response (n:24)	Poor response (n:23)	p value
Pituitary height (mm)	4.29 ± 1.05 (2.40 - 6.50)	4.52 ± 0.88 (2.90 – 6.00)	0.413
Pituitary volume 1 (ellipsoid, mm³)	140.31 ± 68.83 (31.67 - 319.48)	156.33 ± 52.42 (45.91 – 255.46)	0.376
Pituitary volume 1 (SDS)	-0.91 ± 1.02 (-3.81 – 0.50)	-0.30 ± 0.67 (-1.41 – 1.45)	0.021*
Pituitary volume 2 (cross-sectional area, mm³)	184.00 ± 103.67 (37.10 – 470)	198.43 ± 74.23 (42.18 – 325.78)	0.587
Pituitary volume 2 (SDS)	-0.24 ± 1.23 (-3.55 – 2.39)	0.41 ± 1.09 (-1.35 – 2.82)	0.060

*Values in bold represent statistically significant differences (p<0.05).

Data were presented as mean ± standard deviation (min-max).

Good response: HV SDS > 2; Poor response: HV SDS < 2.

## 4 Discussion

In this study, we investigated the relationship between pituitary size and growth response to rhGH therapy in children with isolated GHD and demonstrated that smaller pituitary height and volume were weakly but significantly associated with greater improvements in both height velocity and change in height after one year of treatment. Although pituitary height did not correlate with height velocity or height velocity SDS, our findings suggest that pituitary volume may be associated with the response to GH therapy from an auxological perspective. However, this association may reflect the underlying severity of GH deficiency rather than a direct causal effect of pituitary size on treatment response.

Previous studies have shown that pituitary hypoplasia is frequently associated with more severe GH deficiency, lower peak GH responses in stimulation tests, and lower IGF1 levels (13, 15, 16, 19, 23–26). Consistent with these observations, we found that children with smaller pituitary size exhibited lower IGF1 levels. There are also studies demonstrating an association between pituitary height SDS and peak GH responses (17, 27). However, no significant correlation was observed between pituitary height or volume and baseline height SDS or peak GH responses during L-dopa and clonidine stimulation tests.

Although pituitary hypoplasia is often associated with more severe forms of GHD, it should be noted that GHD may also be present in children with structurally normal pituitary glands, and the diagnosis should be based on a combination of auxological, biochemical, and, where appropriate, radiological findings (14–16).

It is widely accepted that the detection of congenital abnormalities in the hypothalamic–pituitary region on MRI is an important factor in predicting the growth response to rhGH therapy in children with GHD (28, 29). However, the evidence regarding the relationship between pituitary volume and the response to GH therapy remains limited. In a study, pituitary hypoplasia was defined as a pituitary height of less than 3 mm, and patients were categorized into normal and hypoplastic pituitary groups accordingly. After one year of GH therapy, height-for-age Z-scores, the percentage change in height, predicted adult height, growth velocity and growth velocity Z-scores improved significantly in hypoplastic group (14).

In another study where children with GHD were classified according to pituitary height—defined as pituitary hypoplasia for height age, pituitary hypoplasia for chronological age, or normal pituitary height—their response to GH therapy was assessed. The best growth response to GH therapy was observed in patients with pituitary hypoplasia for height age, compared with the other groups (17). Several studies have demonstrated that growth hormone deficient children with hypoplastic pituitary glands respond better to GH therapy (14, 17). However, the classification of pituitary hypoplasia has varied considerably across previous studies. This heterogeneity makes direct comparisons between studies challenging. In contrast, our study evaluated pituitary size using quantitative correlation analyses rather than categorical classifications, allowing a more objective assessment of the continuous relationship between pituitary dimensions and treatment response. Consistent with our findings, a study reported a negative correlation between pituitary volume and both height velocity and height SDS gain after GH treatment. However, no significant association was reported between pituitary height and either height velocity or height SDS gain after therapy (23). The better growth response in children with smaller pituitary size may be due to a congenital, stable defect in somatotroph cell number or function, leading to more pronounced GH and IGF1 deficiency. These patients may therefore demonstrate a better catch-up response.

A very recent study by Oh et al. (30) assessed the association between pituitary volume and growth response to GH therapy. They reported that, among patients with GHD, the pituitary volume in the good-response group—classified by the gain in height SDS during the first year of treatment—was significantly smaller than that of the poor-response group. The authors concluded that pituitary volume may serve as a useful predictive marker for GH treatment response. Similarly, our findings also support that patients classified as good responders (defined by HV SDS > 2) had significantly lower pituitary volume SDS values compared to poor responders.

However, our study adds further value by focusing exclusively on children with isolated GHD, excluding those with idiopathic short stature or organic hypothalamic–pituitary anomalies or multiple hormone deficiencies, thereby reducing etiological heterogeneity. Moreover, we utilized age- and sex-adjusted volumetric SDS derived from national normative data and compared two different volumetric calculation methods, which may offer additional clinical insights not provided by previous studies.

This study has several strengths, including standardized MRI evaluation performed by a single experienced radiologist, the use of two independent methods for calculating pituitary volume, using quantitative correlation analyses and the assessment of auxological and biochemical treatment response parameters. However, certain limitations must be acknowledged. First, the sample size was relatively small, which may have limited the ability to detect stronger associations. Second, the retrospective nature of the study limits our understanding of longitudinal follow-up and long-term response to rhGH therapy. Another limitation of our study is the lack of established age- and sex-specific normative data for pituitary size in our own center. To overcome this limitation, we referenced normative values reported in a previously published study based on a similar Turkish pediatric population (22).

## 5 Conclusions

Our findings indicate that smaller pituitary size is associated with a more favorable growth response to rhGH therapy in children with isolated GHD. Pituitary height and volume may be useful in predicting treatment outcomes. However, it is worth noting that while pituitary volume (measured as ellipsoid formula) SDS showed a significant difference between the two groups, pituitary height alone was not a reliable discriminator of treatment response in our cohort. This finding underscores the potential added value of using ellipsoid volumetric assessment, standardized by age and sex. Rather than suggesting a direct causal relationship between pituitary size and treatment outcome, our findings likely reflect the underlying severity of GHD: children with smaller pituitary glands may have more profound, organic GHD, and thus respond more robustly to GH therapy.

## Data Availability

The raw data supporting the conclusions of this article will be made available by the authors, without undue reservation.
